# Treatment of Patients Undergoing Abdominal Operations1Read before the Erie County Medical Association, June 19, 1904.

**Published:** 1904-08

**Authors:** J. A. MacLeod

**Affiliations:** 274 Delaware Avenue, Buffalo, N. Y.


BUFFALO MEDICAL JOURNAL.

VOL. XLIV.—LX. AUGUST, 1904. No. 1

### ORIGINAL COMMUNICATIONS.

### Treatment of Patients Undergoing Abdominal Operations.[1]

By J. A. MacLEOD, M. D., M. R. C. S. Eng., Buffalo, N. Y.

THE treatment referred to in the foregoing title is undoubtedly as important as the technic of the actual operation itself and it is best discussed under headings.

### THE TREATMENT BEFORE THE OPERATION.

This is principally one of prophylaxis, and its aim is to prepare the patient in such a manner that she will enter the operating theater in the best mental and physical condition possible. The question of shock here is one which needs very careful consideration.

Though not attempting to discuss the pathology of shock, one may say that the condition is due to a state of exhaustion of the medulla and the spinal cord, leading to a great reduction of vital activity and resulting from severe irritation of the peripheral ends of the sensory and sympathetic nerves. In this condition the face is pale and drawn; the pulse is frequent, weak and dicrotic. The pupils are dilated; the reflexes are diminished; the respirations are feeble, irregular and sighing; the temperature is subnormal.

The condition may be much lessened by prophylactic treatment. It is well for the patient to be in the hospital for at least three or four days, even for a week if possible, before the operation, so that she may become accustomed to her surroundings and undergo a careful and systematic preparation.

---

1. Read before the Erie County Medical Association, June 19, 1904.

The patient's mental condition should be considered. All causes of anxiety from outside sources should be guarded against. Her mind should be occupied as much as possible and she should not be allowed to dwell too much on the approaching operation. Her confidence, not only in the operator but also in the anesthetist, should be sought after. Her physical condition is an exceedingly important factor in the case. Each and every organ should be systematically examined. Her general condition should be improved, if necessary, by rest, by tonics, and by hypodermatic injections of strychnine twice or thrice daily for a week before the operation.

Heart lesions if compensated do not necessarily contraindicate operative measures. If there be slight irregularity in the cardiac function it should be treated. The vessel walls should be examined and if thickened, atheromatous, or calcareous, caution should be used.

In kidney lesion, small amounts of albumin do not necessarily contraindicate but copious amounts do in all cases of complacence. The presence of sugar in the urine, if of appreciable amount, is a contraindication in all such operations of complacence. The urine to be examined should always be catheter drawn from female patients to avoid possible contamination by vaginal secretion or discharge. The urine in any case should be passed just before going into the operating theater, and, if necessary, it should be catheter drawn. The blood should be examined and the percentage of the hemoglobin ascertained. In anemic conditions, it is well to put the patient under treatment for some time before operation, as convalescence is much hindered if the hemoglobin be at a low percentage.

The diet during the period of preparation should be regulated and should be light in character. No food at all should be given for four hours before the operation, with the exception of a cup of beef tea two hours before, and some brandy, if necessary, just before going into the theater. Some surgeons give a nutrient enema of beef tea and brandy just before the operation. The mouth should undergo a very careful course of cleansing. The bowels should be regulated by a course of salines, with a stronger purge on the night before, and an enema on the morning of the operation. Magnesium sulphate and magnesium carbonate may be used for the general purpose of clearing out the intestinal tract. If, in addition, an antiseptic effect is required, phosphate of soda, which increases the flow of bile, may be used. Salol and calomel also may be of service where an antiseptic effect is called for. Effervescing salts, for example the seidlitz powder, are usually inferior to the above, and where there is a gastric or intestinal lesion high up they may be the source of danger by inflating the gastrointestinal tube.

The skin should be cleansed by a course of warm baths. The vagina should be daily douched with a mild antiseptic lotion in those cases involving the uterus or its appendages. The patient's clothing for the operation should consist of long woolen stockings, reaching up to the trunk, and of a woolen garment covering the thorax and the upper extremities. In cases where sepsis may be feared antistreptococcic serum, used for a few days previous to the operation, may be of service. The preparation of the actual field of operation belongs rather to the technic of the operation itself and, therefore, I will not deal with it here. Of course in cases of emergency one cannot prepare the patient in such an elaborate way, but even in such cases one should take as much time as possible in the preparation. In cases where there has been a recent severe hemorrhage it may be well to give either an intravenous injection of normal saline solution or a rectal injection of similar fluid. Strychnine may be necessary, and, in fact, may be used as a routine treatment. Where a meal has recently been partaken it may be well to wash out the stomach by use of the stomach tube. The bowels should be evacuated by a copious enema. Then, again, the use of antistreptococcic serum may be of importance.

### THE TREATMENT AND MEASURES TO BE ADOPTED DURING THE OPERATION.

The temperature of the theater should be kept equable and, in some cases where the operation is likely to be a prolonged one, a hot water operating table is of great service. Though not going into the technic of the operation it is well to mention that antisepsis or asepsis should be rigidly carried out. The handling of important viscera should be minimised; undue exposure of parts should be avoided, and careful hemostatic measures should be employed. As to the anesthetist, one skilled in the art of administrating anesthetics should be employed. If the operation be a prolonged one, strychnine or ether given hypodermatically, or rectal injection of normal saline solution may be of great service.

Syncope may occur during the operation and is revealed by pallor, weak or imperceptible pulse, and very shallow breathing. This condition may improve and often terminates in an attack of vomiting. In extreme cases, however, death is the termination. Syncope is very often seen in patients not completely under the influence of the anesthetic and passes off when anesthesia is complete. It is essentially due to anemia of the brain and, therefore, the treatment consists of lowering the head and stimulating the heart.

### THE TREATMENT FOLLOWING THE OPERATION.

Under this heading I will not deal with the question of diagnosis but adhere simply to that of treatment. It is a question that requires much skill and sound judgment and often gives rise to grave anxiety on the part of the man in charge of the case. A majority of cases, when the preceding treatment has been thorough, make an uninterrupted and uneventful convalescence. The treatment in such is simple, and the more simple the better for the patient. A brandy and coffee enema may be given before the patient leaves the theater. She is then transferred to the ward and put gently and carefully to bed, keeping the head low and the foot of the bed slightly raised. She is covered with blankets sufficient to keep her warm, hot water bottles are placed at her feet, legs and to the side of the trunk, care being taken that these bottles are covered with flannel and not hot enough to blister. It may be well to mention here that owing to lowered vitality there is greater danger of blistering in the application of heat than normally.

The room should be dark, well ventilated, kept at an equable temperature, and all noise should be avoided. No food should be allowed for the first twenty-four hours. If the patient complains of thirst, dry tongue and mouth, the latter may be swabbed with a mixture of glycerine and honey. Another efficacious method is to allow the patient to suck a slice of lemon and cracked ice bound up in a bag of thin muslin. If she complains of pain and cannot sleep, a hypodermic of morphine may be allowed. If the bandages are too tight they may be loosened or snipped at the discretion of the surgeon. At the end of twenty-four hours feeding may be started, commencing with very small quantities and gradually increasing in amount, but kept liquid in character until after the bowels have been opened. This may be done by giving an aperient on the second or third night, followed by an enema the next morning. They should then be kept regular thereafter by salines or enemata.

Once the bowels are acting well and regularly the diet may be more generous in character and amount. The bandage when soiled should be changed, care being taken that the dressings are undisturbed. If, however, the dressings are also soiled they should be promptly changed as well. The wound should be dressed on or about the ninth day and at the same time the stitches should be removed. In the above I have not mentioned the drainage tube, but if such has been used the dressings must be changed to meet the requirements of the case. The same may be said if gauze packing has been used.

The patient should be kept in bed for about three weeks, avoiding all unnecessary movement, but not necessarily remaining in one position. The back, buttocks and shoulders should be kept under careful observation. At the end of about three weeks she may be allowed to sit up, but should pass the greater part of the next week on a couch or wheel chair. For the next six months she should avoid doing any heavy work, especially the lifting of any heavy weight. The bowels should be kept regular to avoid any undue straining at stool, and it may be advisable to make use of a low commode, for in so doing the thighs are well flexed on the abdomen.

### COMPLICATIONS OR DEVIATIONS FROM THE NORMAL.

Those cases not making such an exemplary recovery are best discussed, I think, under the separate heading of the complications or deviations from the normal. We may classify these as follows: (1) shock; (2) hemorrhage; (3) vomiting; (4) pain; (5) temperature and pulse variations; (6) thirst; (7) flatulence; (8) intestinal paresis; (9) intestinal obstruction; (10) sepsis; (11) pulmonary complications; (12) pulmonary embolism; (13) phlebitis and thrombosis; (14) fistulæ; (15) bedsores.

*Shock.*—I have stated in the first part of this paper what I consider shock to be, and I have also discussed the prophylactic measures to be adopted in order to avoid the appearance of such a condition. When these measures have been fully carried out shock does not usually play a very important part in the question of after-treatment. In cases where shock was to be feared, either from the gravity of the operation or from its prolonged length, I have found a systematic course of saline rectal injections of great service. The course may be mapped out as follows: a pint and a half of normal saline solution, to which is added one ounce of brandy, are injected immediately on the completion of the operation. The patient then should be put to bed with all the care which I have already described, but the foot of the bed should be raised to a greater height than in the preceding treatment. One pint of normal saline is injected hourly for the next four hours; then every two hours until the pulse is of good volume and tension; and finally every four hours until there are signs of rectal irritation. At the end of twenty-four hours the pulse will usually be nearly normal in rate, volume, and tension. If signs of rectal irritation show themselves early, a suppository of morphine and belladonna usually has the desired effect.

In cases of shock unless the collapse is immediate it is generally delayed for some four to six hours or even more, and under the above regime it usually does not appear at all. If, however, the shock is so great that it appears at once, hypodermic injections of strychnine or ether, or both, may be used, and these may be supplemented by rectal injections of normal saline solution, or by an intravenous injection of saline solution into one of the superficial veins, or by a subcutaneous injection of normal saline into the loose tissues of the breast. In addition to having the foot of the bed raised it is well to bandage the lower extremities with a warm flannel bandage from below upward.

*Hemorrhage.*—This may come on immediately after the operation or, as most frequently, some few hours later when reaction has well set in, or, again, it may be secondary in nature, and may appear at any time after the second day up to the fifth or the sixth day. The reactionary hemorrhage is best prevented by careful technic during the operation, seeing that each and every bleeding vessel is securely tied; the secondary hemorrhage is best prevented by careful asepsis or antisepsis during the operation. Hemorrhage, whenever it does occur, should be carefully watched and if of any magnitude the only treatment is to reopen the abdomen, and secure, if possible, the bleeding point.

*Vomiting.*—This may be due to the anesthetic. Then it is usually simple in character and passes off in a few hours. It is best prevented by careful preparation of the patient before the operation, and by careful and skilful administration of the anesthetic during the operation. It may be relieved by a few doses of iodine, two minims in two drams of water, every two hours. The fumes of vinegar inhaled from a napkin soaked with the liquid are often very efficacious in relieving this early vomiting. When due to the want of proper preparation, washing out the stomach by means of the stomach tube is often of great service. Later on, vomiting may be due to one of the following: (a) intestinal paresis; (b) intestinal obstruction; (c) peritonitis. The treatment of vomiting in each of these complications is essentially that of the condition itself.

*Pain.*—This is sometimes a troublesome complaint especially in neurotic patients, when slight moral suasion may be tried, but when troubling the patient very much morphine may be used hypodermatically at first, and afterward by suppository. The use of morphine, however, should be restricted as much as possible on account of the danger of starting the morphine habit, which often dates from the accidental discovery of the stimulo-sedative power of the drug. I may mention that much discomfort may be prevented by bandaging firmly and evenly up to the level of the ensiform cartilage, thus limiting muscular movement.

*Temperature and Pulse Variations.*—These should be carefully noted every few hours until convalescence has well set in. They are not in themselves complications, but rather symptoms of complications. Their treatment is that of the complication of which they are the symptoms.

*Thirst.*—This may be considerable, but in cases where saline injections are used it is usually not of great importance. The mouth should be swabbed out with a mixture of glycerine and honey, or the patient may be allowed to suck a slice of lemon and cracked ice bound up in a muslin bag. Ice, by itself, is commonly used, the patient sucking it, but I have found the patient does much better if ice in this way is not allowed. Some allow hot water to be sipped, but this is also better omitted.

*Flatulence.*—This may be due to simple causes or it may be caused by (a) intestinal paresis; (b) intestinal obstruction; (c) peritonitis. If it be due to simple causes, it may be treated by small doses of cajuput, peppermint, and the like, or by passing the long rectal tube well up into the sigmoid, leaving it in situ for some time. A stomach tube may be passed into the stomach which very often gives relief. If due to the more serious causes, enumerated above, the treatment is that of the condition of which it is one of the symptoms.

*Intestinal Paresis.*—Though not going into the question of the diagnosis of intestinal paresis, I may say that it is a condition accompanied by constipation, flatulence, temperature and pulse variations, and vomiting. In the treatment of such condition the main object is to procure free action of the bowels. The vomiting usually is so severe that it is not possible to secure this result by the aid of aperients alone. Copious enemata, simple or turpentine, may be used. If these fail it is necessary to stop the vomiting and to this end we have several means at our disposal. We may use one or we may be forced to try the group. Bismuth and hydrocyanic acid diluted may be used, but very often fail; morphine used hypodermatically is frequently successful; cerium oxalate and codeia also may be of service; and sometimes washing out the stomach may produce the desired effect. But the most potent remedy is the hydrochlorate of cocaine used in one eighth grain doses in two drams of water every two hours until the vomiting ceases. The pulse should be carefully watched during the administration of the cocaine and if any untoward signs arise it should be stopped. A hypodermic of strychnine may, however, counteract such adverse signs. The vomiting having ceased, a smart purge should be given, followed in a few hours by a copious enema. The bowels once open should be kept acting freely. In intestinal paresis it is, as a rule, not possible to feed by the mouth and it is then necessary to resort to rectal feeding. Mouth feeding, however, may be started as soon as the bowels are acting freely.

*Intestinal Obstruction.*—This may be due to (a) fecal accumulation; (b) onset of acute peritonitis; (c) causes active in the intestinal wall itself; (d) causes active outside of the intestinal tube. Intestinal obstruction is usually accompanied by signs and symptoms of shock, by pain, by vomiting, and by constipation. Sometimes, however, the lower bowel empties itself and gives rise to false hopes as to the prognosis. If due to fecal accumulations the onset is usually slow and signs of shock may not appear. The treatment is similar to that of intestinal paresis which, in itself, may be practically looked upon as a class of intestinal obstruction. If, however, the obstruction is due to strangulation, be the cause thereof what it may, the onset is sudden and the shock great. The only treatment is to reopen the abdomen which operation must not be too long deferred. It may be necessary to treat the shock before operating. It is wise not to relieve the pain by means of morphine and the like, as symptoms are likely to be masked. The abdomen having been reopened, a search for the cause should be instituted and the obstruction relieved, if possible. It may be necessary to leave the source of trouble alone for the time being and to open the distended bowel above the seat of obstruction. Even if the obstruction be relieved, one should not be satisfied until the bowel is emptied of its fetid contents and, to do this, it may be necessary to open up the lumen of the gut and leave a tube in situ until the acute symptoms have subsided. In urgent cases it may be advisable to operate, especially where fecal vomiting is present, without the aid of a general anesthetic. Such patients are suffering from severe depression and it is found that cocaine used as a local anesthetic is sufficient to meet the requirements of the case.

*Sepsis.*—Though not going into the bacteriology of the question, I may say that it is very important to have any pus that may be found examined microscopically. It is also important to have the blood examined for leukocytosis. Speaking of sepsis in a general way, one may say that it gives rise to temperature and pulse changes, to pain, and to constitutional symptoms. The temperature is usually high and may have gone up suddenly and may have been accompanied by a rigor, but in the extreme cases it is subnormal. In the beginning the pulse is increased in rate, full and bounding, but subsequently it becomes weak, thready, and running; the pain may be great and is due to pressure; the constitutional symptoms are those due to the absorption of toxic material, plus those due to the febrile state which, of course, in itself, is due to the absorption of the toxins. Speaking also of treatment in a general way it is mainly symptomatic. A brisk purge may be given in the initial stages, but later the treatment must be of a more supporting character. Quinine is held by some men to have an effect on pus formation itself, by lessening, or even preventing, its formation. Personally, I do not think that we can expect much from the use of the drug outside of its thermic effect in large doses, and of its tonic effect in small doses. In high febrile conditions quinine is often of great service in depressing the temperature.

The use of serums is, at present, a debatable point, but undoubtedly upon further investigation they will prove to be of great service where the special microörganism, which gives rise to the trouble, can be detected by the microscope. Recently, in the treatment of severe toxemia from peritonitis or other severe inflammations a new mode has arisen—namely, large quantities of normal saline solution are injected intravenously, causing, directly, an increased diuresis and diarrhea, and, indirectly, an increased elimination of toxins. Practically, we may here classify sepsis as to its situation: (a) superficial or wound; (b) deep—localised peritonitis, general peritonitis; (c) general—sapremia, pyemia, septicemia. In the separate treatment of each of the above divisions or subdivisions I will take it for granted that the wound has been exposed and examined.

*Superficial or Wound Sepsis.*—If there be any signs of irritation, or inflammation around any of the stitch holes, the offending stitch or stitches must be removed to relieve tension. If pus has formed, the wound should be opened to the extent of the suppuration and pus evacuated. The wound should then be cleansed, packed, and dressed regularly.

*Deep Sepsis.*—Under this heading may be put the two classes of peritonitis found as complications in abdominal work—namely, localised and general peritonitis. The question of peritonitis is too large a one for me to discuss fully here; let it suffice to say that peritonitis is always due to microörganisms, be their origin what it may.

*Localised Peritonitis.*—This is usually but not necessarily followed by suppuration. It gives rise to the constitutional symptoms of sepsis; vomiting may or may not be present; constipation is usually present. The pain is deep seated and the abdominal muscles over the site of the inflammation are held tense and board like. After suppuration has occurred, the pus may point toward the surface, or it may open into any of the hollow viscera; or, again, it may discharge into the general peritoneal cavity. The treatment in the early stages may be begun with a purge, but later on it should be constitutional and of a supporting character. In some cases it may not be advisable to give a purge, and then the lower bowel should be washed out by an enema. The local lesion should be carefully watched, using at the same time fomentations to relieve the pain. When suppuration has occurred, however, operation is called for. An incision should be made over the tumor and the abscess opened. A gentle search for the cause may be instituted and when found it should be removed, but care should be taken not to break down any of the adhesions shutting off the general peritoneal cavity. The cavity of the abscess should be gently washed out with some mild lotion and free drainage arranged for. It is advisable to wait for 24 or 48 hours before commencing more vigorous irrigation. The case then demands regular dressing.

*General Peritonitis.*—This is accompanied by severe constitutional symptoms, vomiting, constipation, flatulence, distension, severe pain and exquisite tenderness. The temperature at first is usually raised, but towards the end in severe cases it is usually depressed or subnormal. The pulse rate rapidly increases, at first full and bounding but latterly it becomes weak, thready, and running. A falling temperature and an increasing pulse rate is an especially bad omen. Respirations are quick and shallow, being of a thoracic type. This condition having arisen the only treatment that is likely to be of any avail is the early reopening of the abdomen and thoroughly washing it out with normal saline solution. In doing this it may be necessary to expose each and every coil of intestine in order to get at all the pockets of inflammation. The temperature of the lotion should range from 59 to 62 C. Ample provision for drainage should be made before completing the operation. In some cases it may be wise to empty the distended bowel through a trocar puncture which may subsequently be closed by sutures. Where operative measures are not deemed advisable an attempt should be made to obtain a free action of the bowels, and the use of such stimulants as brandy and strychnine should be resorted to freely to support the strength of the patient. Antistreptococcic serum and intravenous injection of normal saline solution may also be given a trial.

*Sapremia.*—This, together with its train of severe constitutional symptoms, is due to the absorption of large quantities of toxins from a focus of suppuration occurring under pressure. The treatment consists of the general constitutional treatment as above depicted, and of local treatment which must be essentially radical in its nature and similar in character to that of deep abscess.

*Pyemia.*—By the term pyemia is meant the formation of multiple abscesses secondary to some focus of local infection. Each abscess gives rise to the signs and symptoms of sepsis. The constitutional symptoms are usually severe. The temperature is of an intermittent type and accompanied by rigors. The general constitutional treatment is as above laid out. The local treatment consists of dealing promptly with each and every focus of inflammation as above described under the treatment of deep abscess.

*Septicemia.*—This is due to the development of microörganisms in the blood and it is accompanied by very severe constitutional symptoms. It is fortunately of rare occurrence in abdominal surgery, but, when it does arise, it is of grave import. The wound should be opened, purified, and provision made for free drainage. Stimulants should be freely resorted to. Antistreptococcic serum in doses of 10 to 15 c.c., twice or thrice daily, may be used. The intravenous injection of normal saline solution may also be given a trial.

*Pulmonary Complications.*—In any elevated condition or sudden rise of the temperature the chest should be carefully examined and if there be any lesion there noted, it should receive prompt treatment. The condition which may be found, and its treatment, belong rather to the field of medicine, and, therefore, I shall not deal with them here.

*Pulmonary Embolism.*—This is due to the impaction of a thrombus, detached in or near the site of the operation, in one of the large pulmonary vessels. The result varies according to the size of the embolus and the situation of the impaction. If small, it may give rise to pain and urgent dyspnea which, however, passes off under cardiac stimulation. Generally, the onset of the trouble is so sudden, and the dyspnea is so great, that death ensues before help can be procured. Prophylaxis is the one important thing. The patient should be kept as quiet as possible, avoiding all unnecessary movement, until any thrombus which may be present is well on to the condition of organisation. The bowels should be kept regular and all straining at stool forbidden. Any bronchitic trouble should receive attention in order to prevent any violent effort in coughing.

*Phlebitis and Thrombosis.*—If these conditions arise the part affected should be purified, wrapped in a sterilised dressing, and kept at absolute rest. If they occur in an extremity, the limb should be slightly elevated. If there be much pain an evaporating lotion or fomentation may be used. The treatment should be continued until organisation is complete.

*Fistulae*—The chief thing in the treatment of fistulæ is prophylaxis in the way of careful technic during the operation. Such a condition having arisen, however, the treatment consists of cleanliness, frequently changing the dressings, syringing the fistulous tract at each dressing with a mild nonirritating lotion, and protecting the skin from the irritating discharges. These fistulæ usually close up in time, but in some cases it is necessary to perform some plastic operation at a later date.

*Bedsores.*—Here, again, prophylaxis is the one important treatment. The under sheet should be kept smooth, straight, and free from creases, and should be frequently drawn. All parts subjected to pressure should be frequently cleansed by the use of some mild soap, and then should be gently rubbed with a spirit lotion, and finally dusted well with boracic powder. If any reddening occurs, the part affected should be protected from further pressure and the reddened surface painted with a mixture consisting of equal parts of tincture of catechu and liquor plumbi subacetatis. When sores actually form they should be cleansed and covered by some mild antiseptic dressing and should be protected from any further pressure.

274 Delaware Avenue.

